# Closed-Incision Negative-Pressure Wound Therapy in Bypass Surgery: Evidence and Implications for Personalized Care

**DOI:** 10.3390/jpm15100448

**Published:** 2025-09-24

**Authors:** Ali Taghizadeh-Waghefi, Veronica De Angelis, Taofeq Bastouni, Stanislaw Vander Zwaag, Manuel Wilbring, Konstantin Alexiou, Klaus Matschke, Utz Kappert, Asen Petrov

**Affiliations:** 1 Medical Faculty “Carl Gustav Carus”, TU Dresden, 01307 Dresden, Germany; 2 Center for Minimally Invasive Cardiac Surgery, University Heart Center Dresden, 01307 Dresden, Germany; 3 Institute of Cardiac Anesthesiology, University Heart Center Dresden, 01307 Dresden, Germany

**Keywords:** sternal wound infection, negative-pressure wound therapy, prevention, coronary artery bypass grafting, personalized medicine, individualized postoperative wound management

## Abstract

**Objectives:** Sternal wound infections (SWIs) after cardiac surgery remain a major complication and represent a significant clinical challenge. This article aims to evaluate the effectiveness of closed-incision negative-pressure wound therapy (ciNPWT) in preventing postoperative wound complications in high-risk patients undergoing coronary bypass surgery via full median sternotomy. **Methods**: Data on all consecutive patients undergoing coronary artery bypass surgery at our facility between March 2021 and March 2023 were retrospectively collected. The ciNPWT group consisted of 71 patients. A control group receiving conventional wound dressings was selected by propensity matching. The primary outcome was postoperative sternal wound complication of any severity, as well as superficial and deep SWIs. The secondary outcomes were hospital stay length, in-hospital mortality, and need for perioperative wound revision. **Results**: The incidence of postoperative SWIs was significantly higher in the ciNPWT group than in the control group (18 [25.4%] vs. 7 [9.9%], *p* = 0.03). Of these 25 cases, 20 had received postoperative ciNPWT and 5 conventional wound dressings, which was statistically different (15 [21.1%] vs. 5 [7.0%], *p* = 0.03). ciNPWT was also significantly associated with positive bacterial cultures (13 [18.3%] vs. 4 [5.6%], *p* = 0.04) and perioperative wound revision (11 [15.5%] vs. 6 [8.5%], *p* = 0.05). **Conclusions**: In consecutive high-risk patients undergoing coronary bypass surgery, the use of prophylactic ciNPWT did not improve wound healing compared to conventional wound dressings, raising concerns about its effectiveness in high-risk patients. Our results do not support the routine use of ciNPWT in this setting. Its potential value may instead lie in carefully defined patient subgroups, underscoring the relevance of our findings for patient-tailored care strategies in cardiac surgery.

## 1. Introduction

Sternal surgical site complications (SSCs) after cardiac surgery represent a significant clinical challenge, as they have been associated with an increased risk of early and late mortality, prolonged hospitalization, and high resource burden for treatment [1,2,3]. Deep sternal wound infections (DSWIs) have been estimated to impact 1.6% of patients after cardiac surgery [4]. DSWIs are associated with significantly elevated rates of short- and long-term adverse clinical outcomes and mortality [4]. Risk factors for sternal SSCs can be classified as patient-related and procedure-related. Patient-related factors include age, body mass index (BMI), smoking, diabetes mellitus, radiation therapy, redo procedures, and chronic lung and kidney diseases. Procedure-related factors include the use of bilateral internal mammary artery grafts during coronary surgery [5].

Across all procedures, DSWIs are most common after coronary artery bypass grafting (CABG) [6,7]. Given the concomitant risk factors often associated with patients undergoing coronary bypass surgery, there is a pressing need for effective strategies to minimize their risk of wound-related issues. Nonetheless, the incidence of DSWIs has remained relatively stable over the past two decades, highlighting the need for increased attention. In this context, the clinical and economic burdens of wound infections have prompted a shift in focus from treatment alone to a greater emphasis on prevention. Therefore, a comprehensive approach to prevention also encompasses intraoperative strategies, including aseptic technique, careful hemostasis, minimal tissue trauma, and the potential use of antibacterial-coated sutures [8].

Initially introduced to assist in the treatment of chronic open wounds, negative-pressure wound therapy (NPWT) has since been adapted for use in closed incisions. Initially adopted for wounds with increased secretions to prevent SSCs, this approach has also been adopted in cardiac surgery to enhance wound healing and prevent complications in high-risk patients.

Closed-incision NPWT (ciNPWT) systems use a negative pressure unit and specific dressings that help to hold the incision edges together, redistribute lateral tension, reduce edema, remove exudate, stimulate perfusion, and maintain a closed environment over the surgical incision, protecting the surgical site from external infectious sources, thereby promoting a favorable environment for wound healing [9,10]. Of the few ciNPWT systems on the market, the Prevena™ Incision Management System (KCI USA, Inc., San Antonio, TX, USA), designed explicitly to manage closed surgical incisions, is currently the most widely used and researched.

In this context, the evaluation of ciNPWT in high-risk CABG patients should also be considered within the broader framework of individualized postoperative wound management, with the aim of tailoring preventive strategies to heterogeneous patient risk profiles rather than applying uniform approaches. This study aimed to evaluate the effectiveness of ciNPWT in preventing postoperative wound complications and determine its potential benefits and limitations in high-risk patients undergoing coronary artery bypass surgery via full median sternotomy.

## 2. Patients and Methods

### 2.1. Study Design and Patients

This retrospective single-center case–control study included all consecutive patients who underwent coronary artery bypass surgery at our facility between March 2021 and March 2023. During the study period, 1683 patients underwent primary, isolated coronary artery bypass surgery. The exclusion criteria were concomitant and redo procedures, surgical approaches other than full median sternotomy, and any interruption or discontinuation of ciNPWT before the sixth postoperative day. Untimely termination of ciNPWT due to external factors (e.g., removal of the device by the patient) led to the exclusion of 11 patients. ciNPWT was used at the discretion of the operating surgeon in patients deemed to be at high risk of SSCs. The ciNPWT group consisted of 71 patients. A control group receiving conventional wound dressings was selected through propensity matching for risk factors, as described below.

### 2.2. Ethical Aspects

The study was approved by the Ethics Committee of the Technical University of Dresden (reference number: EK 298092012, approval date: 18 October 2012) for the retrospective analysis of anonymized patient data from the institutional database.

### 2.3. Study Outcomes

The primary outcome was postoperative sternal SSCs of any severity, as well as superficial SSC and DSWIs. The diagnoses were made and graded according to the U.S. Centers for Disease Control and Prevention criteria. A superficial SSC involves only the skin, subcutaneous tissue, and/or pectoralis fascia without bone involvement. A DSWI required the presence of one of the following:

1. An organism isolated from mediastinal tissue or fluid culture;
2. Evidence of mediastinitis seen during operation; or
3. The presence of chest pain, sternal instability, or fever (>38 °C) and purulent drainage from the mediastinum, or isolation of an organism in blood/mediastinal culture.

### 2.4. Data Collection and Intervention

This retrospective single-center case–control study collected data from the hospital’s database, which was anonymized before completion of the case-report forms. The Prevena™ therapy system (KCI USA, Inc., San Antonio, TX, USA) was used for ciNPWT, which consists of a single-use NPWT unit, canister, and dressing designed for application over clean, closed incisions. ciNPWT was applied immediately after incision closure in the operating theater with continuous negative pressure of −125 mmHg for at least six days. The wounds were inspected after removal of the ciNPWT system before hospital discharge. The patients in the conventional group received conventional sterile gauze dressings, which were first changed on the second postoperative day and then as needed. In cases of suspected wound infection, microbiological samples were obtained.

### 2.5. Follow-Up

Wound status was assessed after ciNPWT removal and before hospital discharge. Demographic, pre-, intra-, and postoperative data were analyzed. The variables included demographics, preoperative risk factors, surgical procedures, perioperative complications, wound-related complications, length of hospital stay, and perioperative survival. Kaplan–Meier curves were used to illustrate the postoperative incidence of wound healing disorders, and the log-rank test was used to evaluate potential differences.

### 2.6. Cost Assessment

In addition to clinical outcomes, a cost analysis was conducted from the hospital perspective limited to the index hospitalization. The analysis included (i) material costs of ciNPWT (Prevena™) The analysis included (i) material costs of ciNPWT (Prevena™) and conventional dressings, and (ii) treatment costs associated with postoperative wound complications observed during the hospital stay. Prices for dressing materials were estimated based on institutional averages (EUR 151.00 per patient for ciNPWT and EUR 1.16 per patient for conventional dressings). Total costs per group and per patient were calculated descriptively. Incremental costs and the incremental cost per additional deep sternal wound infection (DSWI) were derived.

### 2.7. Statistical Analysis

The data were statistically analyzed both descriptively and analytically using the R statistical software (version 4.4.1, R Foundation for Statistical Computing) [11], with a *p*-value of <0.05 considered statistically significant. The normality of each variable’s distribution was assessed using the Shapiro–Wilk test. Continuous variables are expressed as means ± standard deviations (SDs)—compared between groups using Student’s *t*-test if normally distributed—or as medians (interquartile ranges [IQRs])—compared between groups using the Mann–Whitney U test if non-normally distributed. Categorical variables are expressed as counts (percentages) and were compared between groups using the chi-squared test or Fisher’s exact test.

**Figure 1:**
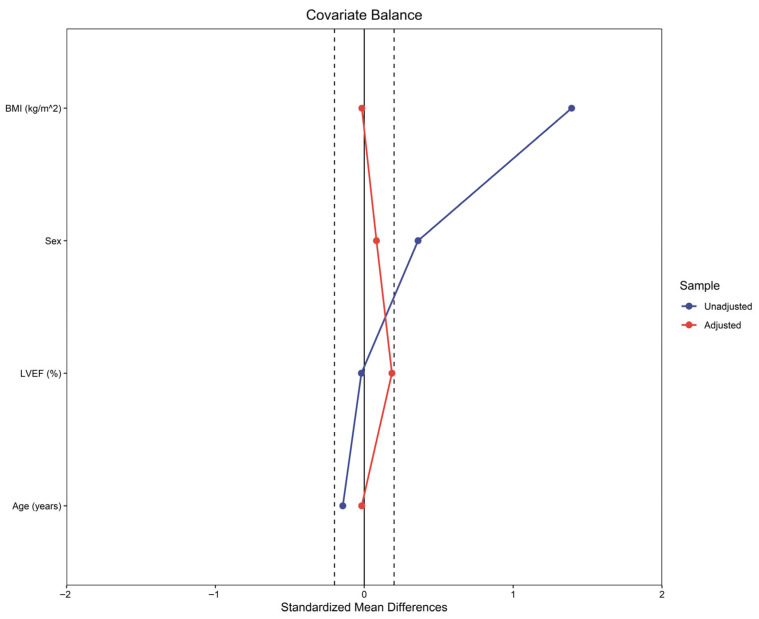
Covariate balance plot before and after propensity-score matching on the selected covariates. BMI: body mass index; LVEF: left ventricular ejection fraction.

To balance baseline characteristics between groups, propensity score matching (1:1, nearest neighbor without replacement) was performed based on age, sex, BMI, and left ventricular ejection fraction (LVEF). The matched standardized differences of the covariates in both cohorts are shown in Figure 1.

To identify independent predictors of DSWIs, univariate logistic regression was first performed. Variables with clinical relevance or significant association in the univariate analyses (*p*-value < 0.1) were then included in a multivariable logistic regression model, and odds ratios (ORs) with 95% confidence intervals (CI) were calculated for each variable.

Kaplan–Meier curves were used to illustrate the postoperative incidence of wound healing disorders, and the log-rank test was used to evaluate potential differences. The impact of wound therapy type (ciNPWT vs. conventional) on the development of postoperative DWSIs was evaluated using univariate logistic regression.

## 3. Results

### 3.1. Clinical Outcomes

The patients’ characteristics and operative variables are summarized in Table 1. Propensity score matching resulted in 71 pairs with similar baseline characteristics. Among the included patients, 69.0% were male, the mean age was 67.0 ± 8.5 years, and the mean BMI was 35.1 ± 4.8 kg/m^2^. The overall incidence of the comorbidities was as follows: diabetes mellitus (56.3%), chronic obstructive pulmonary disease (COPD, 20.4%), peripheral arteriopathy (10.6%), and cerebrovascular arteriopathy (12.7%). The mean incidence of all comorbidities was higher in the study cohort than in the general population undergoing cardiac surgery [12,13]. The patients’ characteristics and preoperative variables did not differ significantly between the ciNPWT and conventional groups, including the European System for Cardiac Operative Risk Evaluation (EuroSCORE) II [14] values (2.09% ± 1.67% vs. 1.96% ± 1.25%, *p* = 0.71).

Procedural characteristics were generally similar between the ciNPWT and conventional groups, with no differences in the rate of use of bilateral mammary arteries, urgency, procedural and cardio-pulmonary bypass times, and type of suture used for wound closure. However, cross-clamp times differed significantly between groups (50.9 ± 16.8 vs. 43.9 ± 15.6 min, *p* = 0.01). The effect of cross-clamp time on the development of DSWIs was evaluated using univariate logistic regression, revealing no significant effect (OR 1.00, 95% CI 0.99–1.00, *p* = 0.09).

The postoperative course was similar in both groups (Table 2), with no significant differences in ventilation times and hospital stay lengths. However, the length of stay in the intensive care unit (ICU) was longer in the conventional group than in the ciNPWT group (2.2 ± 2.4 vs. 2.4 ± 1.6 days, *p* = 0.02). There were no cases of postoperative tracheostomy, renal failure, or in-hospital mortality.

Overall, 25 (17.6%) patients experienced postoperative wound complications, and the incidence was significantly higher in the ciNPWT group than in the conventional group (18 [25.4%] vs. 7 [9.9%], *p* = 0.03). Superficial SSCs were observed in 2 (2.8%) patients in the conventional group and 3 (4.2%) in the ciNPWT group. DSWIs were observed in 5 (7.0%) patients in the conventional group and 15 (21.1%) in the ciNPWT group (*p* = 0.03). These rates were markedly higher than the overall DSWI rate at our facility: Of the total 4935 patients who underwent cardiac surgery via median sternotomy at our facility during the study period, 111 (2.5%) developed postoperative DSWIs. This population encompasses all CABG procedures, valve and aortic surgeries, redo procedures, and operations of any urgency. The much higher rates of wound complications in both the conventional and ciNPWT groups confirm that these are high-risk patients compared to the overall surgical population at our facility.

**Figure 2:**
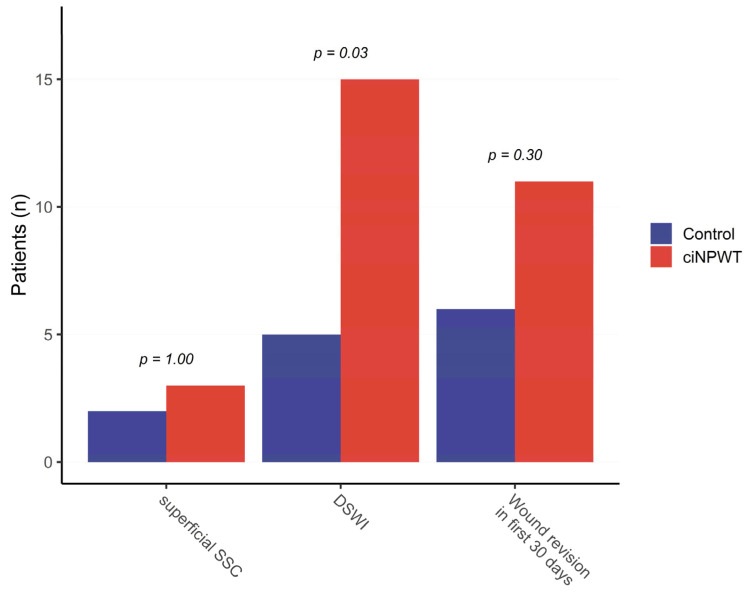
Comparison of postoperative wound complications. ciNPWT: closed-incision negative-pressure wound therapy; DSWI: deep sternal wound infection; SSC: surgical site complication.

The presence of bacterial contamination was confirmed in 13 (18.3%) patients in the ciNPWT group and 4 (5.6%) in the conventional group (*p* = 0.04). The incidence of perioperative wound revision was significantly higher in the ciNPWT group than in the conventional group (11 [15.5%] vs. 6 [8.5%], *p* = 0.05; Figure 2).

**Figure 3:**
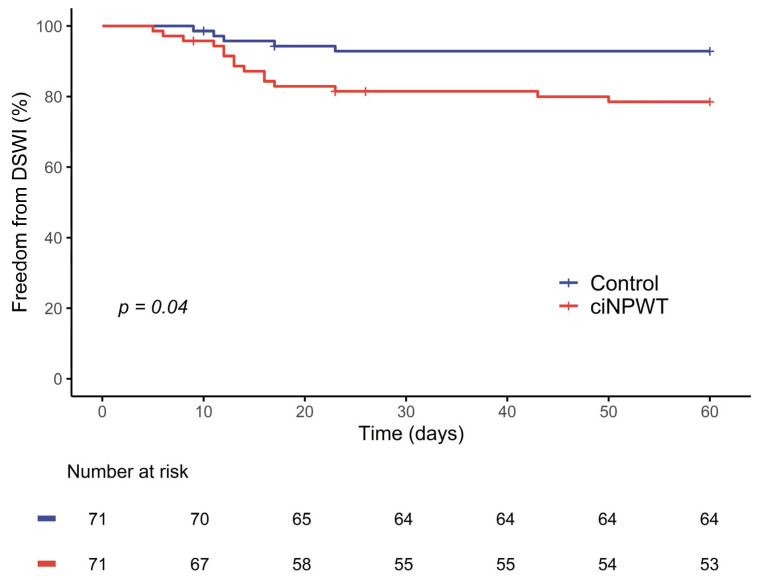
Kaplan–Meier curves showing the estimated freedom from DSWIs (*p* = 0.04, log-rank test). ciNPWT: closed-incision negative-pressure wound therapy; DSWI: deep sternal wound infection.

The estimated freedom from DSWI at 30 days was significantly lower in the ciNPWT group than in the conventional group (83.9% vs. 92.9%, *p* = 0.04; Figure 3). In univariate analysis, several factors were associated with an increased risk of DSWI, including male sex (OR 1.13, 95% CI 1.00–1.28, *p* = 0.04), higher BMI (OR 1.015, 95% CI 1.003–1.027, *p* = 0.02), peripheral arteriopathy (OR 1.24, 95% CI 1.03–1.49, *p* = 0.04), cerebrovascular arteriopathy (OR 1.33, 95% CI 1.13–1.57, *p* < 0.01), and the use of ciNPWT (OR 1.15, 95% CI 1.03–1.29, *p* = 0.02). Cross-clamp time showed a statistical trend towards lower risk (OR 0.997, 95% CI 0.994–1.000, *p* = 0.09).

In the multivariable model including BMI, peripheral arteriopathy, cross-clamp time, and ciNPWT, higher BMI (OR 1.016, 95% CI 1.005–1.027, *p* < 0.01) and peripheral arteriopathy (OR 1.283, 95% CI 1.085–1.518, *p* < 0.01) remained independent predictors of DSWI. Cross-clamp time retained an inverse association (OR 0.997, 95% CI 0.993–1.000, *p* = 0.04). Importantly, ciNPWT also remained independently associated with DSWI (OR 1.115, 95% CI 1.002–1.240, *p* = 0.04) (Table 3).

### 3.2. Cost Analysis

The comparison of direct in-hospital costs revealed a substantial economic disadvantage of ciNPWT. Total costs amounted to EUR 215,238.95 in the ciNPWT group compared with EUR 68,255.01 in the conventional group, corresponding to incremental costs of EUR 146,983.94, or approximately EUR 2070 per patient. The difference was attributable both to higher wound therapy costs related to complications (≈EUR 1920 per patient) and to higher dressing material costs (≈EUR 150 per patient).

## 4. Discussion

The efficacy of ciNPWT in preventing DSWIs, as reported in prior studies, does not appear conclusive [15,16,17,18,19,20,21,22,23,24]. This observation concerning the limited validity of the results of prior studies is supported by a recent systematic review and meta-analysis on the use of NPWT for preventing sternal wound infections in adults after cardiac surgery [25]. The evidence of a beneficial effect of ciNPWT is based on studies with suboptimal quality and a limited number of participants. Moreover, they did not account for selection bias, such as with a prospective randomized design or propensity matching [25]. The issue in determining the actual value of ciNPWT following cardiac surgery lies in part in the rarity of postoperative wound healing disorders in general and DSWIs in particular. Since a sufficient number of events is needed to determine whether a therapy is effective, a large patient cohort would be required for a valid evaluation. Furthermore, an even larger number of participants would be required to identify the subgroup for whom this therapy is most beneficial.

Grauhan et al. examined patients who underwent median sternotomy, reporting significantly different infection rates in the NPWT group (1.3%) compared to the conventional group (3.4%) [21]. Unlike our study, all patients within the study period received ciNPWT (*n* = 237), regardless of the type of procedure or their predisposition for SSCs, resulting in a highly heterogeneous patient group. Despite the large number of patients, comparability may be limited due to heterogeneity and the different time periods of the two study arms: 2013 for the ciNPWT group and 2008–2009 for the conventional group.

Conversely, Myllykangas et al. reported a slightly but nonsignificantly higher incidence of DSWIs and superficial SSCs in the NPWT group, concluding that the routine use of NPWT may not reduce the risk of infections [26]. They included a large series of high-risk patients after CABG and analyzed well-balanced groups after propensity score matching. However, the main limitations of their study were the retrospective nature of the conventional group and the use of the PICO™ ciNPWT system (Smith & Nephew Inc., Andover, MA, USA), which has been shown to provide inferior results compared to the Prevena^TM^ ciNPWT system [26]. Nonetheless, their findings were corroborated by Ruggieri et al., who demonstrated no significant benefit of ciNPWT in a large cohort of propensity-matched patients who underwent bilateral internal mammary artery CABG [27]. The incidence of DSWI/mediastinitis in the ciNPWT group was 5.5%. Notably, patients in our ciNPWT group had markedly higher BMIs (35.3 kg/m^2^) than those in Ruggieri et al. (29.1 kg/m^2^) and Myllykangas et al. (31.4 kg/m^2^), potentially contributing to our higher incidence of DWSIs.

Overall, in our cohort of 142 patients, 17.6% experienced postoperative SSCs, both superficial and deep. Their incidence was significantly higher in the ciNPWT group than in the conventional group (18 [25.4%] vs. 7 [9.9%], *p* = 0.03). Of the 20 cases of DSWIs, significantly more occurred in the ciNPWT group than in the conventional group (15 [21.1%] vs. 5 [7.0%], *p* = 0.03). The ciNPWT group also experienced significantly higher rates of positive bacterial cultures (13 [18.3%] vs. 4 [5.6%], *p* = 0.04) and wound revisions within 30 days (11 [15.5%] vs. 6 [8.5%], *p* = 0.05). We also assessed independent risk factors for DSWI using univariate and multivariable logistic regression. In the univariate analysis, male sex, higher BMI, peripheral arteriopathy, cerebrovascular arteriopathy, and ciNPWT were associated with an increased risk of DSWI, while cross-clamp time showed a statistical trend. In the multivariable model, higher BMI and peripheral arteriopathy remained independent predictors, which is consistent with prior literature [28,29]. Interestingly, cross-clamp time retained an inverse association with DSWI, a counterintuitive finding that is most likely a statistical artifact due to sample size limitations and collinearity with other variables. Importantly, ciNPWT also remained independently associated with higher DSWI risk after adjustment, reinforcing our main observation that this therapy does not appear to provide protection in high-risk CABG patients. Therefore, in our cohort comprised of high-risk patients who underwent isolated coronary artery bypass, ciNPWT failed to achieve its purpose as a primarily preventative measure against postoperative wound healing disorders.

To help interpret our results, we examine the function of ciNPWT. A specific dressing is used to maintain a closed environment over the surgical incision, protecting it from external infectious sources. While standard dressings were usually changed on the second postoperative day and then as needed to assess wound healing, ciNPWT was applied immediately after surgery and left for at least six days, with the wounds only inspected after removal. It has been postulated that most infections are acquired in the operating room [12]; therefore, it can be hypothesized that ciNPWT may protect the surgical wound solely from postoperative contamination and even hinder early detection of superficial SSCs and sternal instability. It also precludes frequent disinfection that occurs with each change of conventional dressings. However, these differences alone cannot explain the exorbitantly high rates of DSWIs in our study cohort. Despite our efforts to minimize selection bias, the designation of patients for ciNPWT by the surgeon may have introduced an unknown confounding variable. Nonetheless, the measurable baseline characteristics were comparable in both groups and do not appear to indicate any fundamental flaws.

Another possible mechanism for developing DWSIs is a purely mechanical sternal instability, which may, for example, be caused by postoperative delirium, which can lead to fractures of the sternal wires or the sternum itself, especially in patients who are obese. Considering the primary mechanism in which ciNPWT functions may further clarify its failure. ciNPWT applies negative pressure to the epidermis of a closed wound incision, thus removing excess fluid, stimulating perfusion, and reducing edema. While this is theoretically beneficial, it might reach its limits, particularly in patients who are obese. In patients with a large amount of presternal fatty tissue, the positive effects of ciNPWT may be limited to the epidermis and uppermost portions of the subcutaneous fat. Even if all the excess fluid is removed from the skin, if fluid is retained subcutaneously and does not reach the incision, no amount of negative pressure will facilitate its removal.

Therefore, the question arises: if ciNPWT promotes wound healing and prevents wound complications in low-risk patients but loses its usefulness in high-risk patients, is it reasonable to use it in the postoperative care of cardiac surgery patients? Low-risk patients experience very low rates of wound complications and would require extensive use of ciNPWT to achieve clinical benefit, which might not justify its costs, whereas high-risk patients might not benefit at all. Therefore, while an all-encompassing prevention of SSCs is highly desirable, ciNPWT does not seem to be the solution currently. Our findings raise concerns about the effectiveness of ciNPWT in high-risk patients and do not support its routine use in this population. Its potential value may instead lie in carefully defined subgroups, which warrants further investigation within the framework of personalized medicine that aims to adapt preventive strategies to individual risk profiles. Beyond clinical considerations, ciNPWT was also linked to higher in-hospital costs compared with conventional dressings, highlighting the importance of thorough evaluation before its broader implementation.

## 5. Limitations

The main limitation of our study was its retrospective, non-randomized design. Therefore, a propensity score analysis was conducted to reduce the effect of selection bias. In addition, our data were collected exclusively from the records of our own facility. Consequently, minor complications that may have been treated at a later stage in other hospitals may be absent from the analysis; however, this limitation applies equally to both groups. Nonetheless, as our facility is the sole tertiary cardiac surgery center with a wide coverage area, patients requiring treatment for postoperative complications are routinely referred back to our facility if needed. Finally, the relatively small number of patients in the study cohort prevents the evaluation of possible confounding factors with small effects. The restricted sample size is since ciNPWT was discontinued in our institution after an interim analysis raised concerns about its benefit, which explains why no further patients were treated with this therapy beyond March 2023.

## 6. Conclusions

In this cohort of high-risk CABG patients, prophylactic ciNPWT did not improve wound healing compared with conventional dressings and was even associated with higher rates of DSWI. While prospective randomized trials are needed to confirm these findings, the routine use of ciNPWT in this population should be approached with caution. Its potential role may lie in selected patient subgroups, which requires further investigation within the framework of personalized medicine.

## Figures and Tables

**Table 1:** Patients’ characteristics and operative variables.

	Overall	ciNPWT	Conventional	*p*-Value
	(*N* = 142)	(*n* = 71)	(*n* = 71)	
Characteristics				
Age (years), mean ± SD	67.0 ± 8.5	67.4 ± 8.9	66.6 ± 8.0	0.58
Sex (male), *n* (%)	98 (69.0%)	48 (67.6%)	50 (70.4%)	0.86
BMI (kg/m^2^), mean ± SD	35.1 ± 4.8	35.3 ± 4.9	34.9 ± 4.8	0.61
EuroSCORE II (%), Mdn (IQR)	1.62 (1.15, 2.39)	1.62 (1.04, 2.56)	1.62 (1.23, 2.29)	0.71
Diabetes mellitus, *n* (%)	80 (56.3%)	43 (60.6%)	37 (52.1%)	0.40
Smoking, *n* (%)	49 (34.5%)	23 (32.4%)	26 (36.6%)	0.72
COPD, *n* (%)	29 (20.4%)	18 (25.4%)	11 (15.5%)	0.21
eGFR (mL/min), mean ± SD	104.0 ± 38.6	108.0 ± 35.1	99.7 ± 41.7	0.06
Dialysis, *n* (%)	1 (0.7%)	0 (0%)	1 (1.4%)	1.00
Peripheral arteriopathy, *n* (%)	15 (10.6%)	10 (14.1%)	5 (7.0%)	0.28
Cerebrovascular arteriopathy, *n* (%)	18 (12.7%)	10 (14.1%)	8 (11.3%)	0.80
LVEF (%), Mdn (IQR)	54.0 (45.0, 60.0)	54.0 (46.5, 60.0)	54.0 (45.0, 60.0)	0.33
Operative variables				
Procedural urgency, *n* (%)				
- Elective	63 (44.4%)	35 (49.3%)	28 (39.4%)	0.43
- Urgent	75 (52.8%)	34 (47.9%)	41 (57.7%)	
- Emergency	4 (2.8%)	2 (2.8%)	2 (2.8%)	
Double internal mammary artery, *n* (%)	8 (5.6%)	5 (7.0%)	3 (4.2%)	0.72
Skin to skin time (min), mean ± SD	172 ± 34.4	174 ± 32.8	170 ± 36.0	0.48
CPB time (min), mean ± SD	62.3 ± 18.4	64.3 ± 19.3	60.4 ± 17.5	0.21
Cross clamp time (min), mean ± SD	47.3 ± 16.5	50.9 ± 16.8	43.9 ± 15.6	0.01
Type of wound suture, *n* (%)				
- Intracutaneous	16 (11.3%)	9 (12.7%)	7 (9.9%)	0.61
- Transcutaneous	1 (0.7%)	1 (1.4%)	0 (0%)	
- Staples	125 (88.0%)	61 (85.9%)	64 (90.1%)	

Mdn: median; BMI: body mass index; ciNPWT: closed-incision negative-pressure wound therapy; COPD: chronic obstructive pulmonary disease; CPB: cardio-pulmonary bypass; eGFR: estimated glomerular filtration rate; LVEF: left ventricular ejection fraction.

**Table 2:** Postoperative variables.

	Overall	ciNPWT	Conventional	*p*-Value
	(*N* = 142)	(*n* = 71)	(*n* = 71)	
Postoperative variables				
Ventilation time (hours), Mdn (IQR)	5.00 (4.00, 7.75)	5.00 (4.00, 8.00)	5.00 (4.00, 7.00)	0.33
Surgical site complication, *n* (%)	25 (17.6%)	18 (25.4%)	7 (9.9%)	0.03 *
- No SSC	117 (82.4%)	53 (74.6%)	64 (90.1%)	
- Superficial SSC	5 (3.5%)	3 (4.2%)	2 (2.8%)	
- DSWI	20 (14.1%)	15 (21.1%)	5 (7.0%)	
Ascertained infection, *n* (%)	17 (12.0%)	13 (18.3%)	4 (5.6%)	0.04 *
Wound revision, *n* (%)				
- None	121 (85.2%)	56 (78.9%)	65 (91.5%)	0.05 *
- ≤30 days	17 (12.0%)	11 (15.5%)	6 (8.5%)	
- >30 days	4 (2.8%)	4 (5.6%)	0 (0%)	
ICU stay (days), Mdn (IQR)	1.00 (1.00, 3.00)	1.00 (1.00, 2.00)	2.00 (1.00, 3.00)	0.02 *
Hospital stay (days), Mdn (IQR)	8.00 (5.00, 11.0)	8.00 (6.50, 10.0)	8.00 (7.00, 11.0)	0.34
In-hospital mortality, *n* (%)	0 (0%)	0 (0%)	0 (0%)	1.00

Note: Bold and italic values indicate statistical significance: * *p* ≤ 0.05. Abbreviations: Mdn, median; ciNPWT, closed-incision negative-pressure wound therapy; DSWI: deep sternal wound infections; SSC, surgical site complication; ICU, intensive care unit.

**Table 3:** Univariate and multivariate analysis.

Variables	Deep Sternal Wound Infection			
	Univariate Analysis		Multivariate Analysis	
	OR (95% CI)	*p*	OR (95% CI)	*p*
Patients’ characteristics				
Age	0.998 (0.992, 1.005)	0.67	-	
Sex (male)	1.133 (1.002, 1.282)	0.04 *	-	
Body mass index (kg/m^2^)	1.015 (1.003, 1.027)	0.02 *	1.016 (1.005, 1.027)	<0.01 **
EuroSCORE II (%)	1.019 (0.980, 1.060)	0.34	-	
Hypertension	1.017 (0.792, 1.306)	0.90	-	
Diabetes	1.021 (0.909, 1.147)	0.72	-	
Dyslipidemia	0.941 (0.835, 1.062)	0.33	-	
Smoking	0.972 (0.861, 1.097)	0.65	-	
Immunosoppressive therapy	0.865 (0.611, 1.224)	0.41	-	
COPD	1.040 (0.902, 1.200)	0.59	-	
eGFR (mL/min)	1.000 (0.999, 1.002)	0.81	-	
Dialysis	0.868 (0.436, 1.728)	0.69	-	
History of wound infection	1.439 (0.886, 2.339)	0.14	-	
Peripheral arteriopathy	1.240 (1.032, 1.491)	0.02 *	1.283 (1.085, 1.518)	<0.01 **
Cerebrovascular arteriopathy	1.328 (1.125, 1.569)	<0.01 **	-	
NYHA Class III or IV	1.049 (0.927, 1.186)	0.45	-	
Left ventricular ejection fraction (%)	1.003 (0.997, 1.008)	0.34	-	
Operative variables				
Procedural urgency	0.968 (0.862, 1.087)	0.59	-	
Double internal mammary artery	0.861, (0.672, 1.105)	0.24	-	
Skin to skin time (min)	1.000, (0.998, 1.001)	0.6	-	
Cardio-pulmonary bypass time (min)	0.999, (0.996, 1.002)	0.38	-	
Cross clamp time (min)	0.997, (0.994, 1.000)	0.09 *	0.997 (0.993, 1.000)	0.04 *
ciNPWT	1.151, (1.028, 1.289)	0.02 *	1.115 (1.002, 1.240)	0.04 *

Note: Bold and italic values indicate statistical significance: * *p* ≤ 0.05; ** *p* ≤ 0.01. Abbreviations: OR, odds ratio; CI, confidence interval; eGFR, estimated glomerular filtration rate; COPD, chronic obstructive pulmonary disease; NYHA, New York Heart Association; ciNPWT, closed-incision negative-pressure wound therapy.

## Data Availability

The data presented in this study are available on request from the corresponding author. The data are not publicly available due to ethical regulations.

## Abbreviations

The following abbreviations are used in this manuscript:

BMI	Body mass index
CABG	Coronary artery bypass grafting
CI	Confidence interval
ciNPWT	Closed-incision negative-pressure wound therapy
COPD	Chronic obstructive pulmonary disease
CPB	Cardio-pulmonary bypass
DSWI	Deep sternal wound infection
eGFR	Estimated glomerular filtration rate
EuroSCORE II	European System for Cardiac Operative Risk Evaluation II
ICU	Intensive care unit
LVEF	Left ventricular ejection fraction
NPWT	Negative-pressure wound therapy
OR	Odds ratio
SSC	Surgical site complication
SWI	Sternal wound infections

## References

[1] Coskun D., Aytac J., Aydinli A., Bayer A. (2005). Mortality rate, length of stay and extra cost of sternal surgical site infections following coronary artery bypass grafting in a private medical centre in Turkey. J. Hosp. Infect..

[2] Gaudino M., Audisio K., Rahouma M., Robinson N.B., Soletti G.J., Cancelli G., Masterson Creber R.M., Gray A., Lees B., Gerry S. (2023). Association between sternal wound complications and 10-year mortality following coronary artery bypass grafting. J. Thorac. Cardiovasc. Surg..

[3] Kaspersen A.E., Nielsen S.J., Orrason A.W., Petursdottir A., Sigurdsson M.I., Jeppsson A., Gudbjartsson T. (2021). Short- and long-term mortality after deep sternal wound infection following cardiac surgery: Experiences from SWEDEHEART. Eur. J. Cardiothorac. Surg..

[4] Perezgrovas-Olaria R., Audisio K., Cancelli G., Rahouma M., Ibrahim M., Soletti G.J., Chadow D., Demetres M., Girardi L.N., Gaudino M. (2023). Deep Sternal Wound Infection and Mortality in Cardiac Surgery: A Meta-analysis. Ann. Thorac. Surg..

[5] Song Y., Chu W., Sun J., Liu X., Zhu H., Yu H., Shen C. (2023). Review on risk factors, classification, and treatment of sternal wound infection. J. Cardiothorac. Surg..

[6] Sharif M., Wong C.H.M., Harky A. (2019). Sternal Wound Infections, Risk Factors and Management—How Far Are We? A Literature Review. Heart Lung Circ..

[7] Hirahara N., Miyata H., Motomura N., Kohsaka S., Nishimura T., Takamoto S. (2020). Procedure- and Hospital-Level Variation of Deep Sternal Wound Infection from All-Japan Registry. Ann. Thorac. Surg..

[8] Pogorelic Z., Stricevic L., Elezovic Baloevic S., Todoric J., Budimir D. (2024). Safety and Effectiveness of Triclosan-Coated Polydioxanone (PDS Plus) versus Uncoated Polydioxanone (PDS II) Sutures for Prevention of Surgical Site Infection after Hypospadias Repair in Children: A 10-Year Single Center Experience with 550 Hypospadias. Biomedicines.

[9] Dohmen P.M., Markou T., Ingemansson R., Rotering H., Hartman J.M., van Valen R., Brunott M., Segers P. (2014). Use of incisional negative pressure wound therapy on closed median sternal incisions after cardiothoracic surgery: Clinical evidence and consensus recommendations. Med. Sci. Monit..

[10] Smolle M.A., Nischwitz S.P., Hutan M., Trunk P., Lumenta D., Bernhardt G.A. (2020). Closed-incision negative-pressure wound management in surgery—Literature review and recommendations. Eur. Surg..

[11] The R Core Team (2025). A Language and Environment for Statistical Computing.

[12] Clough R.A., Leavitt B.J., Morton J.R., Plume S.K., Hernandez F., Nugent W., Lahey S.J., Ross C.S., O’Connor G.T. (2002). The effect of comorbid illness on mortality outcomes in cardiac surgery. Arch. Surg..

[13] Scrutinio D., Giannuzzi P. (2008). Comorbidity in patients undergoing coronary artery bypass graft surgery: Impact on outcome and implications for cardiac rehabilitation. Eur. J. Cardiovasc. Prev. Rehabil..

[14] Nashef S.A., Roques F., Sharples L.D., Nilsson J., Smith C., Goldstone A.R., Lockowandt U. (2012). EuroSCORE II. Eur. J. Cardiothorac. Surg..

[15] Jennings S., Vahaviolos J., Chan J., Worthington M.G., Stuklis R.G. (2016). Prevention of Sternal Wound Infections by use of a Surgical Incision Management System: First Reported Australian Case Series. Heart Lung Circ..

[16] Witt-Majchrzak A., Zelazny P., Snarska J. (2015). Preliminary outcome of treatment of postoperative primarily closed sternotomy wounds treated using negative pressure wound therapy. Pol. Przegl Chir..

[17] Tabley A., Aludaat C., Le Guillou V., Gay A., Nafeh-Bizet C., Scherrer V., Bouchart F., Doguet F. (2020). A Survey of Cardiac Surgery Infections with PICO Negative Pressure Therapy in High-Risk Patients. Ann. Thorac. Surg..

[18] Suelo-Calanao R.L., Thomson R., Read M., Matheson E., Isaac E., Chaudhry M., Loubani M. (2020). The impact of closed incision negative pressure therapy on prevention of median sternotomy infection for high risk cases: A single centre retrospective study. J. Cardiothorac. Surg..

[19] Colli A., Camara M.L. (2011). First experience with a new negative pressure incision management system on surgical incisions after cardiac surgery in high risk patients. J. Cardiothorac. Surg..

[20] Elhassan H., Amjad R., Palaniappan U., Loubani M., Rose D. (2024). The negative pressure wound therapy for prevention of sternal wound infection: Can we reduce infection rate after the use of bilateral internal thoracic arteries? A systematic literature review and meta-analysis. J. Cardiothorac. Surg..

[21] Grauhan O., Navasardyan A., Tutkun B., Hennig F., Muller P., Hummel M., Hetzer R. (2014). Effect of surgical incision management on wound infections in a poststernotomy patient population. Int. Wound J..

[22] Grauhan O., Navasardyan A., Hofmann M., Muller P., Stein J., Hetzer R. (2013). Prevention of poststernotomy wound infections in obese patients by negative pressure wound therapy. J. Thorac. Cardiovasc. Surg..

[23] Rashed A., Csiszar M., Beledi A., Gombocz K. (2021). The impact of incisional negative pressure wound therapy on the wound healing process after midline sternotomy. Int. Wound J..

[24] Brega C., Calvi S., Albertini A. (2021). Use of a negative pressure wound therapy system over closed incisions option in preventing post-sternotomy wound complications. Wound Repair. Regen..

[25] Biancari F., Santoro G., Provenzano F., Savarese L., Iorio F., Giordano S., Zebele C., Speziale G. (2022). Negative-Pressure Wound Therapy for Prevention of Sternal Wound Infection after Adult Cardiac Surgery: Systematic Review and Meta-Analysis. J. Clin. Med..

[26] Myllykangas H.M., Halonen J., Husso A., Vaananen H., Berg L.T. (2022). Does Incisional Negative Pressure Wound Therapy Prevent Sternal Wound Infections?. Thorac. Cardiovasc. Surg..

[27] Ruggieri V.G., Olivier M.E., Aludaat C., Rosato S., Marticho P., Saade Y.A., Lefebvre A., Poncet A., Rubin S., Biancari F. (2019). Negative Pressure versus Conventional Sternal Wound Dressing in Coronary Surgery Using Bilateral Internal Mammary Artery Grafts. Heart Surg. Forum.

[28] de Tymowski C., Provenchere S., Para M., Duval X., Grall N., Sahnoun T., Iung B., Kerneis S., Lucet J.C., Montravers P. (2025). Deep sternal wound infection after cardiac surgery: A combination of 2 distinct infection types, deep incisional surgical-site infection and mediastinitis: Results of a retrospective study. Surgery.

[29] Gundestrup L., Florczak C.K., Riber L.P.S. (2023). Factors associated with deep sternal wound infection after open-heart surgery in a Danish registry. Am. Heart J. Plus.

